# Supplementary material for: Effect of Long-Term Strontium Exposure on the Content of Phytoestrogens and Allantoin in Soybean
Source: Int J Mol Sci. 2018 Dec 4;19(12):3864. doi: 10.3390/ijms19123864 (PMC6321324; doi:10.3390/ijms19123864)
Supplement: Supplementary file 1 [file ijms-19-03864-s001.pdf]

Supplementary material

Effect of Long-Term Strontium Exposure on the Content of Phytoestrogens and Allantoin in Soybean

Sławomir Dresler [1,*], Magdalena Wójciak-Kosior [2,*], Ireneusz Sowa [2], Maciej Strzemski [2], Jan Sawicki [2], Jozef Kováčik [3], and Tomasz Blicharski [4]

1 Department of Plant Physiology, Institute of Biology and Biochemistry, Maria Curie-Skłodowska University, Akademicka 19, 20-033 Lublin, Poland

2 Department of Analytical Chemistry, Medical University of Lublin, Chodźki 4a, 20-093 Lublin, Poland; irek.sowa@gmail.com (I.S.), maciej.strzemski@poczta.onet.pl (M.S.), 91chem91@gmail.com (J.S.)

3 Department of Biology, University of Trnava, Priemyselná 4, 918 43 Trnava, Slovak Republic; jozkovacik@yahoo.com

4 Orthopaedics and Rehabilitation Clinic, Medical University Lublin, Chodźki 4a, Lublin 20-093, Poland; tomasz.blicharski@umlub.pl

* Correspondence: slawomir.dresler@poczta.umcs.lublin.pl (S.D.); kosiorma@wp.pl (M.W.-K.); Tel.: +48-81-537-5078 (S.D.); +48-81-448-7180 (M.W.-K.)

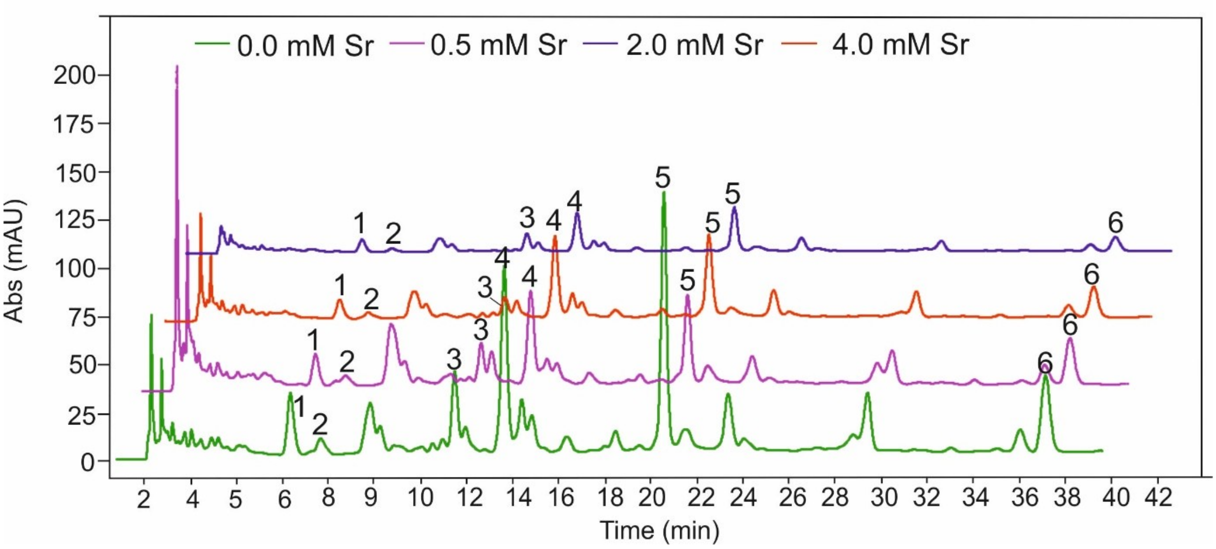

Suppl. Figure 1. Example of an HPLC chromatogram of soyabean leaves extracts treated with different Sr concnetrations; **1**-daidzin, **2**-glycitin, **3**-genistin, **4**-malonyldaidzin, **5**-malonylgenistin, **6**-coumestrol.

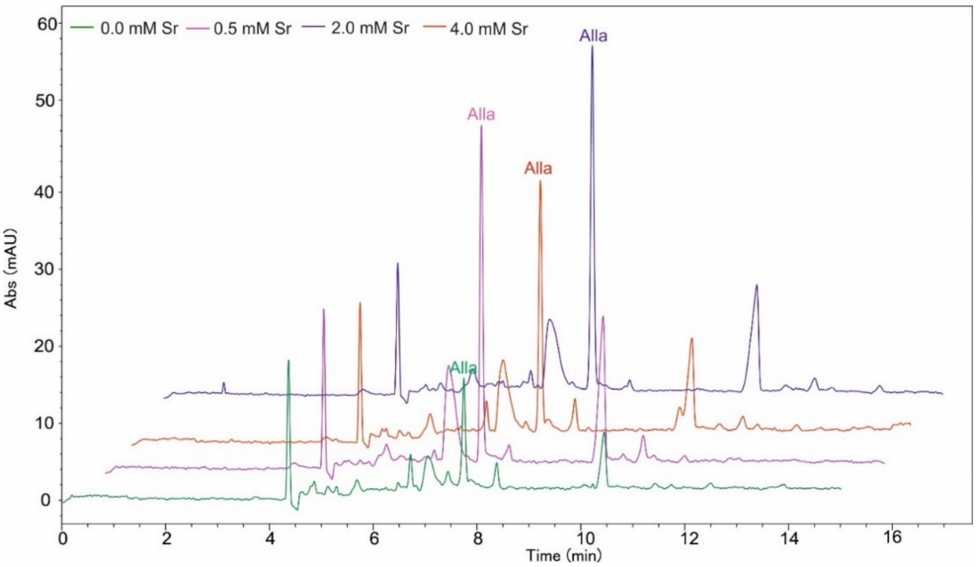

Suppl. Figure 2. Example of an electrophoregram of soybean roots extracts treated with different Sr concentrations (Alla – allantoin).

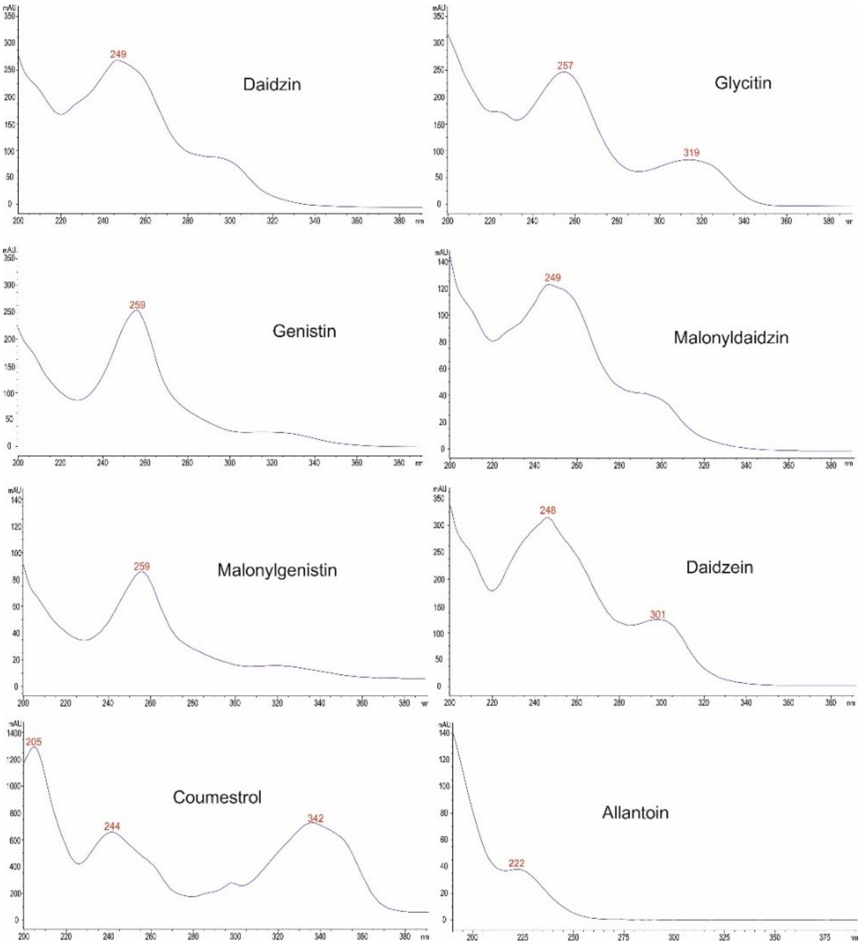

Suppl. Figure 3. UV-Vis spectrum of studied compounds.

Table S1. Effect of various strontium concentrations on the content of zinc, nickel, copper, and manganese ($\mu g \cdot g^{-1}$ ADW) in different soybean organs. Data are means ±SE (n=4). Values followed by the same letters within the same plants organ are not significantly different ($p<0.05$, Tukey's test).

**Leaves**	**Zn**	**Ni**	**Cu**	**Mn**

0.0 Sr	31.6 ± 11.4 a	0.64 ± 0.19 a	6.02 ± 0.29 a	12.66 ± 2.41 a
0.5 Sr	61.1 ± 6.6 a	1.09 ± 0.15 a	7.12 ± 0.11 a	11.06 ± 0.20 a
2.0 Sr	35.5 ± 4.9 a	0.94 ± 0.08 a	6.96 ± 0.41 a	9.84 ± 0.66 a
4.0 Sr	47.4 ± 9.0 a	0.94 ± 0.04 a	7.08 ± 0.15 a	12.71 ± 1.27 a
**Stems**				
0.0 Sr	29.1 ± 9.4 a	0.58 ± 0.04 b	6.51 ± 0.16 b	2.31 ± 0.12 b
0.5 Sr	49.9 ± 13.9 a	0.87 ± 0.12 ab	6.91 ± 0.33 b	3.75 ± 0.61 ab
2.0 Sr	34.5 ± 7.2 a	1.13 ± 0.09 a	7.30 ± 0.32 ab	3.52 ± 0.18 ab
4.0 Sr	23.7 ± 2.3 a	0.89 ± 0.06 ab	8.76 ± 0.05 a	5.07 ± 0.57 a
**Seeds**				
0.0 Sr	81.3 ± 9.1 a	1.22 ± 0.11 a	11.61 ± 1.46 a	8.22 ± 1.59 a
0.5 Sr	85.0 ± 8.4 a	1.19 ± 0.15 a	11.98 ± 2.14 a	7.91 ± 1.02 a
2.0 Sr	83.9 ± 9.0 a	1.12 ± 0.07 a	12.19 ± 0.44 a	6.94 ± 0.79 a
4.0 Sr	96.7 ± 5.3 a	1.29 ± 0.17 a	11.67 ± 1.46 a	5.10 ± 0.63 a
**Roots**				
0.0 Sr	36.2 ± 2.8 a	0.93 ± 0.18 c	29.64 ± 0.73 c	8.27 ± 0.47 a
0.5 Sr	38.1 ± 3.1 a	1.52 ± 0.04 bc	25.93 ± 0.75 bc	7.07 ± 0.31 a
2.0 Sr	31.2 ± 5.1 a	2.50 ± 0.37 ab	41.70 ± 4.64 a	8.61 ± 0.87 a
4.0 Sr	36.1 ± 4.6 a	2.41 ± 0.18 a	38.38 ± 2.74 ab	9.07 ± 1.49 a
